# A randomized controlled trial of the Transdiagnostic Intervention for Sleep and Circadian dysfunction implemented via facilitation and delivered by community mental health providers: Improving the “fit” of psychological treatments by adapting to context

**DOI:** 10.21203/rs.3.rs-5422372/v1

**Published:** 2024-12-24

**Authors:** Allison G. Harvey, Emma R. Agnew, Rafael Esteva Hache, Julia M. Spencer, Marlen Diaz, Estephania Ovalle Patino, Anne Milner, Lu Dong, Amy M Kilbourne, Daniel J. Buysse, Catherine A. Callaway, Laurel D. Sarfan

**Affiliations:** University of California Berkeley; University of California Berkeley; University of California Berkeley; University of California Berkeley; University of California Berkeley; University of California Berkeley; University of California Berkeley; RAND Corporation; Veterans Health Administration; University of Pittsburgh; University of California Berkeley; University of California Berkeley

**Keywords:** facilitation, community mental health, mental illness, sleep, circadian, insomnia, transdiagnostic, psychosis, depression, anxiety disorder, bipolar disorder

## Abstract

**Background.:**

To determine if the use of theory, data and end-user perspectives to guide an adaptation of the Transdiagnostic Intervention for Sleep and Circadian Dysfunction (TranS-C) yields better outcomes and improves the “fit” of TranS-C to community mental health centers (CMHCs), relative to the standard version.

**Methods.:**

Ten counties in California were cluster-randomized by county to Adapted or Standard TranS-C. Within each county, adults who exhibited sleep and circadian dysfunction and serious mental illness (SMI) were randomized to immediate TranS-C or Usual Care followed by Delayed Treatment with TranS-C (UC-DT). Facilitation was the implementation strategy. The participants were 93 CMHC providers who delivered TranS-C (Standard = 30; Adapted = 63) and 396 CMHC patients (Standard = 74; Adapted = 124; UC-DT = 198). Patient assessments were completed at pre-treatment, post-treatment, and six months after treatment (6FU). Provider assessments were completed at post-training, mid-treatment, and post-treatment.

**Results.:**

TranS-C (combining Adapted and Standard), relative to UC-DT before delayed treatment with TranS-C, was associated with improvement from pre- to post-treatment in sleep disturbance (*b* = −10.91, *p* < 0.001, *d* = −1.52), sleep-related impairment (*b* = −9.52, *p* < 0.001, *d* = −1.06), sleep health composite (*b* = 1.63, *p* < 0.001, *d* = 0.95), psychiatric symptoms (*b* = −6.72, *p* < 0.001, *d* = −0.52), and overall functional impairment (*b* = −5.12, *p* < 0.001, *d* = −0.71). TranS-C’s benefits for functional impairment and psychiatric symptoms were mediated by improvements in sleep and circadian problems. Adapted versus Standard TranS-C did not differ on provider ratings of fit and better fit did not mediate the relation between TranS-C condition and patient outcome.

**Conclusions.:**

TranS-C can be delivered by CMHC providers. Although Adapted and Standard TranS-C both fit the CMHC context, several advantages emerged for the adapted version.

## Background

A critical barrier to expanding access to evidence-based psychological treatments (EBPTs) is the potential mismatch between research settings, in which EBPTs are typically developed, and routine practice settings, in which EBPTs are ultimately meant to be delivered. This can result in a poor “fit” between the EBPT and the routine practice setting. Strong fit is defined as the “match between the strategies, procedures, or elements of an intervention and the values, needs, skills, and resources available in a setting” (p. 1) (1). Fit is crucial because there is evidence that many routine practice settings that initially adopted EBPTs cannot sustain them, in part due to poor fit (e.g., 2, 3). Indeed, EBPT fit directly predicts a range of implementation (4) and sustainment outcomes (e.g., 5, 6) and is often included in implementation science frameworks (e.g., 7). Thus, to expand access to EBPTs, it is critical to develop procedures to maximize fit to routine practice settings. Given the multitude of settings globally, the procedures to improve fit must be replicable and generalizable. As a “platform” for evaluating one approach to enhancing fit, we selected one context and one EBPT as exemplars for potential future broader applications.

The context for this project was a network of community mental health centers (CMHCs). Fit is particularly important for resource-constrained practice settings, such as CMHCs. In the United States, CMHCs are large providers of affordable mental health services for people who are low-income and diverse with respect to demographic and clinical presentations. CMHC providers have insufficient time and resources, carry a heavy caseload, and the patients served experience high rates of comorbidity and complexity (8–10). Also, it can be difficult for CMHC providers to receive adequate training and supervision in EBPTs (11).

The EBPT for this project was the Transdiagnostic Intervention for Sleep and Circadian Dysfunction (TranS-C) (12). TranS-C is grounded in, and aims to improve, each dimension of the Sleep Health Framework (13). TranS-C targets the sleep and circadian dysfunction most frequently experienced by people diagnosed with serious mental illness (SMI). Sleep and circadian dysfunction predicts and precedes the onset and worsening of SMI symptoms, as well as poorer mental (e.g., 14, 15) and physical health (e.g., 16), and is modifiable (e.g., 17, 18, 19). In a prior trial, Standard TranS-C relative to usual care, was associated with sustained improvements in sleep and circadian problems, functional impairment, and psychiatric symptoms in CMHCs (18). However, the providers of TranS-C for that study were employed, trained, and supervised within an academic setting. Following the National Institute of Health’s Stage Model, for the present study, we took the critical next step; namely, we tested TranS-C in community settings with *CMHC providers* delivering the intervention (20).

Chambers et al. (21) has cautioned against “creating and freezing an intervention” as this reduces the customization and optimization that are the basis of effective sustainment (p. 2). How might an EBPT be customized and optimized to fit a specific context? The answer most certainly involves theory, data, and end-user input (18, 22, 23). As a demonstration of the use of these elements to conduct a treatment adaptation, we devised a version of TranS-C that was adapted to improve the fit between the CMHC context and TranS-C. In doing so, we sought to empirically test the implementation strategy of “promoting adaptability” (24). In other words, does this strategy of promoting adaptability—defined as “identify[ing] the ways a clinical innovation can be tailored to meet local needs and clarify[ing] which elements of the innovation must be maintained to preserve fidelity”—enhance implementation and clinical outcomes? The treatment resulting from this process will be referred to as “Adapted TranS-C”. As described in the protocol paper (25), the Replicating Effective Programs (REP) framework (26) was selected as the theoretical basis for the adaptation process. Phase 1 of REP (Pre-Condition) was completed prior to the present study. This involved several elements. First, we established that there is a need for an effective, feasible EBPT for SMI in CMHCs and that sleep and circadian functioning was a target that could help address this need (18). Second, we established that TranS-C in CMHCs has empirical support (18). Third, we used the implementation strategy of identifying barriers and facilitators to gather perspectives from CMHC staff on the fit and packaging of TranS-C (27, 28). In this process, both the dose and complexity of Standard TranS-C were considered to be barriers to the implementation of TranS-C (28). Fourth, a prior study identified the TranS-C treatment skills that were most utilized by patients. (29) The associated treatment elements were retained as fixed components of Adapted TranS-C. Fifth, we considered TranS-C’s theoretical underpinnings and mechanisms of action. (12, 13) Treatment strategies that addressed the key mechanisms were also retained as fixed components (23, 30, 31). Sixth, we piloted Adapted TranS-C with 21 adults through the PI’s UC Berkeley research clinic (unpublished data). Feedback from the providers and the patients were used to further refine Adapted TranS-C. In Phase 2 of REP (Pre-Implementation), based on feedback from CMHC leadership, staff, and patients, we tailored the delivery of TranS-C training and therapy materials to the CMHC setting (27, 28). Throughout REP Phases 1 and 2, following leading adaptation frameworks, we sought to ensure that Adapted TranS-C would be relevant to the broadest range of patients and to account for factors that impact implementation (e.g., resources required) (23, 32, 33). Collectively, these efforts addressed Phases 1 and 2 of the REP framework. The current study aims to address Phase 3 (Implementation) of REP. Subsequent reports from other parts of the larger study will address Phase 4 (Maintenance and Evolution) of REP (34, 35). Following prior research in CMHCs (36), Adapted TranS-C involved a modular design, with 5 fixed core modules that address the key mechanisms of change. There is also one optional module that is delivered only when a patient is experiencing sleep-related worry. Given the CMHC context, the general strategy was to remove all excess treatment elements to provide the most simple and efficient version of TranS-C. Beyond these fixed components, providers in both treatment arms were told that they could make fidelity-consistent ‘soft’ adaptations in how they delivered the fixed intervention components in ways that felt relevant and accessible for patients (37). For instance, instead of using examples offered in the treatment manual, providers were encouraged to use examples that were relevant to their specific client. As another example, if the language in the treatment manual seemed too complex for a given client, providers were encouraged to simplify using their own wording.

The overarching goal of this study was to evaluate whether manipulating the fit of TranS-C to the CMHC context (Adapted TranS-C) predicts better implementation outcomes relative to an original version of TranS-C (Standard TranS-C). This study is a hybrid type 2 effectiveness-implementation study (38) conducted in the CMHCs of counties across California in the United States. This report is focused on Parts 1 of three parts. Part 1 is the Implementation Phase, during which TranS-C was implemented in CMHCs via facilitation (25). Parts 2 and 3 will be reported in subsequent papers. Part 2, is the Train-the-Trainer Phase, during which CMHC providers learn to train and supervise their peers in the delivery of TranS-C (34). Part 3 is the Sustainment Phase, during which we will assess the extent to which TranS-C activities are sustained after facilitation has ceased (35).

As described elsewhere (25), all CMHC sites were cluster-randomized by county to Adapted TranS-C or Standard TranS-C with 1:1 allocation. Then, within each county, patients were randomized to immediate TranS-C or UC-DT. TranS-C was delivered by CMHC providers.

For the present study, the first aim was to assess the effectiveness of TranS-C, compared to UC-DT. Note that the assessment points for the latter were completed before and after usual care and before delayed treatment with TranS-C. We hypothesized that compared to UC-DT, TranS-C (combined Adapted and Standard) would be associated with larger reductions in the primary patient outcome of sleep disturbance and the secondary patient outcomes of sleep-related impairment, overall sleep health, functional impairment, and psychiatric symptoms. We also hypothesized that TranS-C’s benefits for functional impairment and psychiatric symptoms would be mediated by improvements in sleep and circadian problems. The second aim was to evaluate whether TranS-C treatment condition (Adapted versus Standard TranS-C) is associated with fit to the CMHC context, operationalized as provider ratings of acceptability, feasibility, and appropriateness. We hypothesized that Adapted TranS-C would be superior to Standard TranS-C with respect to the primary provider outcome of acceptability and the secondary provider outcomes of feasibility and appropriateness. The third aim was to evaluate whether better fit mediates the relation between TranS-C treatment condition and patient outcome. We hypothesized that relative to Standard TranS-C, Adapted TranS-C would be associated with greater reductions in the primary and secondary patient outcomes indirectly through higher provider ratings of acceptability, feasibility, and appropriateness. Exploratory analyses sought to: (1) compare Adapted and Standard TranS-C on patient perceptions of credibility/improvement, and select PhenX Toolkit outcomes; and (2) determine whether treatment effects are moderated by risk factors including age, sex, sleep symptoms, impairment and psychiatric symptoms at baseline (e.g., 39).

## Method

### Setting and Participants

Community health center sites in the following ten counties in California, USA participated: Alameda, Contra Costa, Kings, Monterey, Placer, Santa Cruz, Solano, Santa Clara, Santa Barbara, and Lake. The participants were 93 CMHC providers (Standard TranS-C = 30; Adapted TranS-C = 63) and 396 CMHC patients^1^. Of the patients, 198 were randomized to receive TranS-C immediately (Standard TranS-C = 74; Adapted TranS-C = 124) and 198 were randomized to UC-DT. The larger number of participants in Adapted TranS-C resulted from stronger recruitment in the counties cluster randomized to this condition, as compared to the Standard TranS-C condition. The inclusion criteria for selecting the CMHC sites within counties from which to recruit providers and patients were: (1) provision of publicly funded adult mental health outpatient services and (2) support from CMHC leadership.

CMHCs preferred to determine which providers were eligible to receive TranS-C training at each site (e.g., case managers, nurses, psychiatrists), because this aligns with their real-world practice. The other inclusion criteria for providers were: (1) employed or able to deliver client-facing services to patients within the CMHC; (2) interested in learning and delivering TranS-C; and (3) volunteered to participate and formally consent to participate.

The inclusion criteria for patients were: (1) aged 18 years and older; (2) met criteria for an SMI per self-report and confirmed by referring provider or administration of the Mini International Neuropsychiatric Interview (MINI; DSM-5, Version 7.0.0) by a licensed clinical social worker on the research team^2^; (3) exhibited a sleep or circadian disturbance as determined by endorsing 4 (quite a bit) or 5 (very much), or the equivalent for reverse scored items, on one or more PROMIS-Sleep Disturbance questions (40, 41); (4) guaranteed place to sleep for at least two months that was not a shelter; (5) received the standard of care for the SMI and consent to regular communications between the research team and provider; and (6) consented to access their medical record and participate in assessments.

Patients were excluded if they met any of the following criteria: (1) presence of an active and progressive physical illness or neurological degenerative disease directly related to the onset and course of the sleep and circadian problems, or that made participation in the study unfeasible, as assessed by the Checklist of Medical Conditions and Symptoms on the Duke Structured Interview for Sleep Disorders (42) and clinical interview; (2) presence of substance abuse/dependence only if it made participation in the study unfeasible; (3) current active intent or plan to commit suicide (those with suicidal ideation were eligible) only if it made participation in the study unfeasible, or homicide risk; (4) night shift work for more than two nights per week in the past three months (i.e., regularly scheduled work from 12 a.m. – 6 a.m.); or (5) pregnant or breastfeeding.

### Facilitation

As described in the protocol paper (25) and in Additional File 1, facilitation was selected as the core implementation strategy based on promising evidence (e.g., 43, 44, 45). Facilitation refers to the “multi-faceted interactive process of problem solving, enabling and supporting individuals, groups, and organizations in their efforts to adopt and incorporate innovations into routine practices” (p. 46). It is grounded in the Promoting Action on Research Implementation in Health Services (PARiHS) framework (47). In the present study, each CMHC received direct support from the lead facilitator, who is a licensed clinical social worker with expertise in community mental health and sleep treatment (ERA), and a team of trained facilitators employed by the research team. Throughout the study, the facilitation team was supervised by the Principal Investigator (PI; AGH) with periodic check-ins with a REP and facilitation expert (AMK). Facilitation activities were also informed by the Veterans Affairs facilitation manual (48) and Harvey and Kitson’s (49) Facilitation Guide. Additionally, the lead facilitator (ERA) and postdoctoral scholar (LDS) completed the Behavioral Health Veterans Affairs Quality Enhancement Research Initiative Implementation (BH QUERI) Facilitation Training and regularly attended BH QUERI’S monthly drop-in consultation group. Day-to-day facilitators conducted ongoing assessments at each CMHC site and planned responses to address unmet needs and reduce barriers via an integrated set of evidence-based implementation strategies (44, 50).

The facilitators completed an Implementation Log weekly for 17 months, yielding almost 4,000 hours of facilitation-related activities. Knowing that the processes and strategies involved in implementation are complex and not always described in research reports, presenting barriers to replication and translatability, the log was designed to track a comprehensive range of key implementation variables in near ‘real time’. The log was developed following Proctor et al.’s (51) reporting guidelines for research on implementation strategies. All variables for the log were derived through piloting, collaboration with the facilitators, and/or based on standardized frameworks, taxonomies, and guidelines from the implementation science literature. In addition, qualitative analyses using deductive and inductive coding were used to analyze data from a semi-structured interview that assessed facilitator perceptions of the log with respect to acceptability, appropriateness, and feasibility (51). The first manuscript reporting the results of the Implementation Log is currently “in preparation” (52).

### Interventions

Two variations of TranS-C were tested: Standard TranS-C and Adapted TranS-C. Both were delivered alongside the usual care offered by each CMHC. The control condition was UC-DT. In the CMHCs, usual care consists of working with a service provider (e.g., psychologist, case manager, occupational therapist, psychiatrist, nurse practitioner) who provides direct mental health support alongside other services as needed (e.g., housing support).

Although most providers delivered TranS-C via individual sessions, some opted to deliver it in a group setting. Note that TranS-C was originally developed in English, then translated into Spanish to expand access. TranS-C was offered by 18 Spanish-speaking providers to 35 Spanish-speaking patients.

### Standard TranS-C

Standard TranS-C was delivered by CMHC providers across eight 50-minute, weekly sessions (Harvey & Buysee, 2017). It was comprised of 4 cross-cutting modules featured in every session, 4 core modules, and 7 optional modules used based on clinical presentation, treatment goals, and provider case conceptualization (see Additional File 1 for description). Training for the Standard TranS-C condition consisted of a 1-day workshop (i.e., 6–8 hours) or two, 3-hour training blocks, based on CMHC preference.

### Adapted TranS-C

We grounded the process for adapting TranS-C in theory, data, and end-user input (see Additional File 1 for further details). Adapted TranS-C was delivered by CMHC staff across four, 20-minute, weekly sessions (see Additional File 1 for description). Treatment consisted of the same four *cross-cutting modules* as in Standard TranS-C as well as three of the core modules and one of the optional modules. Training for the Adapted TranS-C condition consisted of four, 1-hour workshops or two, 2-hour workshops, based on CMHC preferences.

### UC-DT

In UC-DT, patients began with usual care for four or eight weeks, depending on whether their CMHC was randomized to Adapted TranS-C or Standard TranS-C. After the delay, they received Adapted or Standard TranS-C, similarly based on the condition to which their CMHC had been randomized. The decision to include UC-DT as the comparison condition was made based on advice from the early CMHC partners to strike a balance between (a) including a comparison group to demonstrate the effectiveness of TranS-C in community settings; (b) ensuring that *all* participants receive what we hypothesize to be an active treatment (TranS-C); and (c) maximizing efficiency in terms of study duration, budget, and participants’ time investment. Notably, usual care has been the comparison group in several influential studies (36, 53).

### Measures

In addition to the measures below, a sociodemographics form was completed by providers and patients.

### Providers

#### Primary Outcome.

##### Acceptability.

Providers rated the acceptability of TranS-C via the *Acceptability of Intervention Measure* (AIM) (Weiner et al., 2017). This 4-item measure was rated on a scale from 1 (completely disagree) to 5 (completely agree). This measure has demonstrated satisfactory validity, internal reliability, test-retest reliability, and sensitivity to change (54).

#### Secondary Outcomes.

##### Appropriateness and Feasibility.

Providers rated the appropriateness and feasibility of TranS-C via the following 4-item measures: *Feasibility of Intervention Measure* (FIM) and *Intervention Appropriateness Measure* (IAM) (54) using the same scale as the AIM.

#### Other Measures.

##### Number of TranS-C Sessions.

The number of sessions delivered to each enrolled patient by each provider was counted.

##### Occupation.

Providers were asked to report their current position, professional degree, and work history, including their caseload, theoretical orientation, licensure status, and previous training in sleep treatment.

### Patients

#### Primary Outcome.

##### Sleep Disturbance.

The 8-item PROMIS-Sleep Disturbance (PROMIS-SD) assessed disruption to sleep (e.g., trouble staying asleep) over the past seven days. Items were rated on a scale from 1 (not at all/never/very poor) to 5 (very much/always/very good). T-scores were used (Yu et al., 2011), calculated by summing the raw scores and using conversion tables on healthmeasures.net, where higher scores indicate more severe symptoms. This measure has demonstrated acceptable reliability and validity (40, 41).

#### Secondary Outcomes.

##### Sleep-Related Impairment.

The 8-item^3^ PROMIS-Sleep Related Impairment (PROMIS-SRI) assessed daytime impairment related to sleep problems using the same scale, timeframe an scoring as the PROMIS-SD.

##### Functional Impairment.

Functional impairment was assessed via the Sheehan Disability Scale (SDS) (55). Impairment in work and school, social life, and home and family was rated via three items on a scale from 0 (not at all) to 10 (extremely). Scores ranged from 0–30, with higher scores indicating greater impairment. This measure demonstrated good reliability and validity (55).

##### Overall Sleep Health.

The Sleep Health Composite captures overall sleep health for the complexity of sleep problems in SMI that are covered by TranS-C (56). It is defined as the sum of scores on six sleep health dimensions (each dimension dichotomized as 1 = good; 0 = poor): Regularity (midpoint fluctuation), Timing (mean midpoint), Efficiency (sleep efficiency), Duration (total sleep time), Satisfaction (sleep quality question on PROMIS-SD), and Alertness (daytime sleepiness question on PROMIS-SRI). All dimensions – except Satisfaction and Alertness – were assessed via questions about sleep-wake patterns over the past seven days. Scores ranged from 0–6, with higher scores indicating better sleep health. Initial validity of this measure has been established (56).

##### Psychiatric Symptoms.

The DSM-5 Cross-Cutting Measure assessed psychiatric symptoms across 13 mental health domains. Participants rated how often they were bothered by each symptom on a scale from 0 (not at all) to 4 (nearly every day). Scores ranged from 0–52, with higher scores indicating more severe symptoms. This measure has demonstrated good test-retest reliability and clinical utility (57, 58).

#### Exploratory Outcomes.

##### PhenX Toolkit.

(59) (see Additional File 2 for further details). To assess suicidal ideation and behaviors, two subscales from the screening version of the Columbia-Suicide Severity Rating Scale—Severity of Suicidal Ideation and Suicidal Behavior—were used. Ideation was assessed in the past month and suicidal behavior in the past three months. These scales were scored according to the scoring guide (60). For ideation, the highest numerical value (i.e., the value associated with the most severe item endorsed, ranging from 1 to 5) was used as the final score. For suicidal behavior, five suicide-related behaviors were assessed by separate items, scored with a binary scale (0 = no, 1 = yes) and frequency of patients who endorsed a given behavior was identified. The PhenX ‘Alcohol – 30-Day Quantity and Frequency’, ‘Tobacco – 30 Day Quantity and Frequency’, ‘Substances – 30-Day Frequency’, and ‘Supplemental Beverage Questionnaire’ were used to assess alcohol, tobacco, psychoactive substance, and caffeine consumption over the past 30 days.

##### Credibility and Perceived Improvement.

Perceptions of TranS-C credibility and symptom improvement were assessed by four questions adapted from the Credibility/Expectancy Questionnaire (CEQ) (Devilly & Borkovec, 2000). These questions assessed (1) how logical TranS-C seemed, (2) how successful it was in reducing sleep symptoms, (3) how confident patients would be in recommending TranS-C to a friend, and (4) how much improvement patients believe had occurred. All questions were rated on a scale from 0 (not at all) to 9 (very), except for perceived improvement, which was rated as a percentage from 0–100%.

##### UC-DT Contamination.

At the end of UC-DT and before starting TranS-C, we assessed for patient exposure to TranS-C during the UC-DT waiting period by asking “Have you received any sleep intervention, treatment, or coaching since entering the study?” If yes, the assessor asked for details. Two independent coders rated the responses for potential exposure to TranS-C.

### Procedure

CMHCs and patients were randomized through a computerized randomization sequence. When randomizing patients, we stratified for presence of psychosis or not (current), presence of substance use or not (current) and age (≥ 50 or not), as there was evidence these variables can impact sleep or treatment outcome (39, 61, 62). Only the facilitators, assessors, and research team (i.e., not CMHCs, providers, or patients) were privy to which CMHCs and patients were allocated to which TranS-C treatment condition (Adapted versus Standard TranS-C). CMHC providers and patients knew whether a patient had been randomized to receive the immediate or delayed treatment. Facilitation was selected as the core implementation strategy used to implement TranS-C as described above. The launch of this study coincided with the COVID-19 pandemic. Thus, most study processes were conducted virtually. The methods described below have been published, often in more detail, in the protocol paper (25).

Providers and patients were consented by the assessment team prior to participation. All patients were compensated for their participation, and providers were compensated if permitted by their CMHC. The assessments were completed by the assessment team, comprised of experienced assessors. Because the assessors needed to provide study-related information—such as number of assessments and treatment sessions—to the patients during the consent process, the assessors were not blind to condition at pre-treatment. However, at post-treatment and the 6FU, we endeavored to keep assessors blind to condition. Assessors received ongoing supervision and were thoroughly trained to deliver the surveys with integrity and minimal bias.

For Aim 1, the patient assessments for the immediate TranS-C condition were completed at pre-treatment and post-treatment. In UC-DT, the patient assessments for Aim 1 were completed at pre-treatment and four or eight weeks after pre-treatment (i.e., at the end of usual care and before delayed treatment with TranS-C), depending on whether their county had been randomized to Adapted or Standard TranS-C, respectively. We did collect a 6-month follow-up (6FU) for immediate TranS-C but we did not include these in the analyses because the prespecified analyses for Aim 1 focus on timepoints with a comparable UC-DT comparison group. For Aim 2, provider assessments of acceptability, feasibility, and appropriateness were measured at post-training, mid-treatment, and post-treatment. Following the prespecified analyses, only the change from post-training to post-treatment was examined because change at post-treatment was the primary effect of interest, and we sought to minimize risks of additional comparisons. For Aim 3 and exploratory Aim 1, as delineated in the prespecified analyses, both post-treatment and 6FU were included for immediate TranS-C to examine effects of TranS-C treatment condition (Standard vs. Adapted TranS-C) on patient outcomes, because (a) assessing change to post-treatment and 6FU (i.e., immediate and sustained change) were both high priority and (b) comparable comparisons for Adapted and Standard TranS-C were available at both timepoints. For exploratory Aim 2, treatment effects were examined only from pre- to post-treatment, because as with Aim 1, comparable comparisons between TranS-C and UC-DT were only available at these timepoints.

Information on the recruitment of CMHCs, providers and patients is available in Additional File 2.

### Trial Registration, Data Transparency and Openness

All research materials, data, and analysis code are available from the authors upon request. This study was preregistered on clinicaltrials.gov (identifier: NCT04154631), a protocol paper was published (25) and the study received approval from the Committee for the Protection of Human Subjects at the University of California, Berkeley. Updates made to the clinicaltrials.gov protocol (identifier: NCT04154631) in December, 2022 are summarized in the protocol paper. Since then, in March 2023 we (a) clarified that the protocol covered the Implementation Phase and not the other phases, (b) we separated the entry for the Utilization Questionnaire into two entries, one for post-treatment and the other for 6FU and (c) we clarified the timeframes for several measures.

### Power Analysis

A pre-specified power analysis was conducted for the entire trial, which included providers and patients from the implementation phase (i.e., CMHC providers trained by the UC Berkeley team and the focus of the present study) (25) and the train-the-trainer phase (i.e., CMHC providers who were trained by local trainers, who had been trained by the UC Berkeley team) (34). However, these two phases were subsequently separated to more thoroughly investigate results from each phase of the study (i.e., implementation phase and train-the-trainer phase). Thus, for the present study, the minimum detectable effect sizes (MDES) were calculated with the sample from the implementation phase for the primary outcomes: sleep disturbance for patients and acceptability for providers. Optimal Design software for cluster-randomized trials with repeated measurements was used to calculate the MDES (63, 64). The intraclass correlation coefficients (ICC) were calculated for multilevel models with timepoints (level 1) nested within patients or providers (level 2) nested with CMHCs (level 3). The resulting ICCs were very small (< 0.0001). To be conservative, an ICC of 0.001 was used. For sleep disturbance, using this ICC of 0.001, the final sample size of *N* = 396 patients, alpha of 0.05, 10 CMHCs, and power = 0.80, the MDES was 0.42. Based on the effect sizes yielded for the main patient-level analyses, this MDES was exceeded. For acceptability, using this ICC, the final sample size of *N* = 93 providers, alpha of 0.05, 10 CMHCs, and power = 0.80, the MDES was 0.96. To account for the possibility that the provider analyses may have been underpowered, significance values and effect sizes are emphasized for providers.

### Analysis Plan

Analyses generally followed the plan specified in the protocol paper (25). Deviations from this plan have been noted in the sections below. Information about assumption checks, missing data and covariates are included in the Additional File 3 and Additional File 4, Tables 1, 2 and 3.

### Analyses

All analyses were conducted with Stata Version 16.1.

#### Multilevel Models (Aims 1 & 2 and Exploratory Aims 1 & 2).

For Aims 1 & 2 and Exploratory Aims 1 & 2, multilevel models (MLMs) were used to account for multiple observations nested within patient (65). Analyses used intent-to-treat principles and maximum likelihood estimation, which performs well in simulations of MLMs with missing data up to 50% (66). All models used robust standard errors. Effect sizes for all multilevel models are represented with ‘*d*’ and were calculated following Feingold (67, Eq. 5), using unadjusted change scores and raw standard deviations at pre-treatment from each treatment condition. The Benjamini-Hochberg procedure (68) was used to correct for multiple testing on the primary outcomes (sleep disruption and acceptability), per the protocol paper. All MLMs compared pre-treatment to post-treatment and, for level 1, included a dummy-coded time indicator as the predictor (1 = post-treatment, pre-treatment as the reference). Exploratory Aim 1 also compared pre-treatment to six-month follow-up and included an additional time indicator accordingly. The level 2 equation included dummy-coded treatment condition (*Aim 1 and Exploratory Aim 2*: 1 = immediate TranS-C, with UC-DT as the reference; *Aim 2 and Exploratory Aim 1*: 1 = Adapted TranS-C, with Standard as reference) and treatment-by-time interaction terms, which were the parameters of interest. Additionally, Exploratory Aim 2 included three-way interactions between time, treatment, and the following pre-specified moderators: sex (dummy coded: 0 = male, 1 = female), age (dummy coded: 0 = < 50, 1 = ≥ 50, and continuous baseline variables of PROMIS-SD, PROMIS-SRI, SDS, and DSM-5 Cross-Cutting. As part of the prespecified analyses for Exploratory Aim 1, linear regression models were also used to test the effects of TranS-C treatment condition on credibility and perceived improvement at post-treatment. The predictor was dummy-coded TranS-C treatment condition (1 = Adapted TranS-C, with Standard as reference) and the outcomes were credibility, expectancy, and total CEQ. Effect sizes for linear regressions are partial eta squared, or the proportion of variance explained by the predictor of interest (69).

Almost all outcomes were continuous, except for the following binary outcomes tested in Exploratory Aim 1: suicidal thoughts and behaviors and illicit substance use. For these outcomes, multilevel logistic regression was used. However, because few participants endorsed these items, the models would not converge. Instead, the frequencies of patients’ endorsement of each item are presented in Additional File 4, Table 4.

We list the outcomes included in each MLM (see Additional File 3 for covariates and below for SEMs). For the Aim 1 MLMs, the outcomes were PROMIS-SD, PROMIS-SRI, Sleep Health Composite, DSM-5 Cross-Cutting, and SDS. Note that, in the protocol paper, the Sleep Health Composite was listed in the Measures section but was omitted from the planned analysis section in error. For Aim 2, the outcomes were providers’ perceptions of acceptability, feasibility, and appropriateness. For Exploratory Aim 1, the MLM outcomes were severity of suicidal ideation, average cigarettes per day among people who endorsed using tobacco, average number of caffeinated drinks per day, and number of days the patient consumed alcohol in the past 30 days. As noted above, the linear regression outcomes Exploratory Aim 1 were credibility, expectancy, and total CEQ. For Exploratory Aim 2, the outcomes mirrored Aim 1 and were PROMIS-SD, PROMIS-SRI, Sleep Health Composite, DSM-5 Cross-Cutting, and SDS.

#### Structural Equation Modeling (SEM) (Aims 1 & 3).

For the mediation models in Aims 1 and 3, SEM was used. Specifically, the analysis of covariance (ANCOVA) approach was used, in which pre-treatment measures of the mediator and outcome are included as covariates.^4^ This approach has been recommended for designs comparing pre- to post-treatment (70). In particular, statisticians and methodologists have argued that contemporaneous models, whereby the mediators and outcomes are both measured at post-treatment, may confer advantages for clinical trials, because these timepoints capture the interval during which the greatest changes are most likely to occur in the mediators and outcomes (e.g., 70, 71, 72). For Aim 1, the predictor was condition (immediate TranS-C vs. UC-DT), the mediator was PROMIS-SD or PROMIS-SRI at post-treatment, and the outcomes were DSM-5 Cross-Cutting and SDS at post-treatment. For Aim 3, the predictor was TranS-C condition (Adapted vs. Standard), the mediator was AIM, FIM, or IAM at post-treatment, and the outcomes were PROMIS-SD, PROMIS-SRI, DSM-5 Cross-Cutting, and SDS at post-treatment and six-month follow-up. For all SEMs, the parameter of interest was the indirect effect. Maximum likelihood estimation was used. As noted above, all models were run with robust standard errors. Effect sizes for mediation models are the mediated proportions (MP), or the proportion of the total effect that is explained by the indirect effect expressed as a percentage (73).

## Results

**Figure 1:**
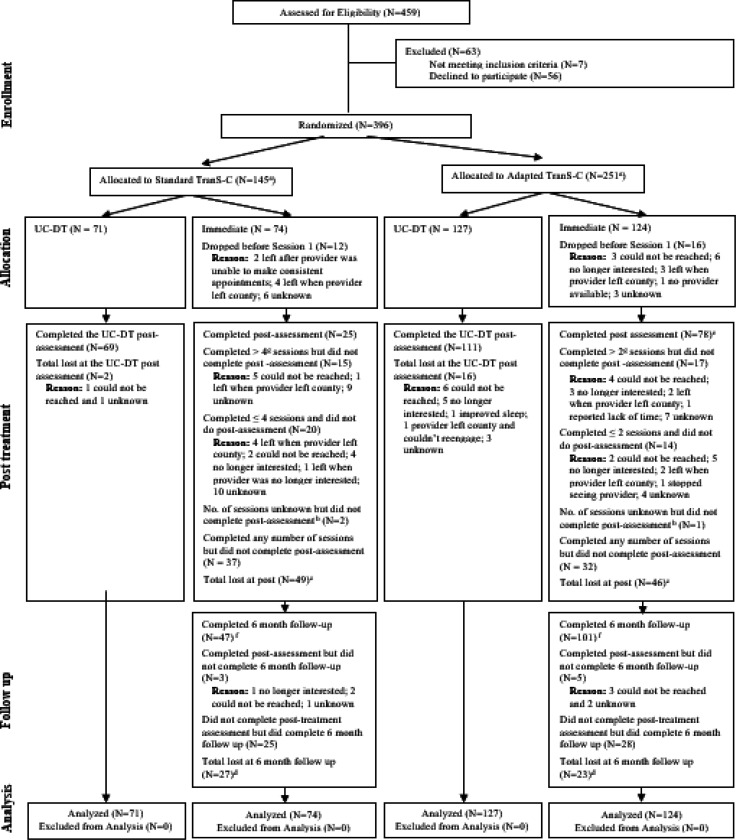
CONSORT Diagram Illustrating the Flow of Patients Through the Study *Note.* ^a^The larger N in Adapted vs. Standard TranS-C was a result of stronger recruitment in the counties randomized to this condition. ^b^We could not determine the count of sessions completed when we when we lost contact with provider and client. ^c^Total lost at post is calculated by summing those who dropped before session 1 and those who completed any number of sessions but did not complete a post-assessment. In the immediate condition the 2 participants who completed a post-assessment who dropped before session 1 are subtracted from this sum. ^d^Total lost at 6-month follow up is calculated by subtracting those who completed follow-up from the initial N. ^e^Out of 78 who completed post-assessment, 2 dropped *before* Session 1. ^f^6-month follow-up was 6 months from the end of treatment. ^g^Drop out is defined as completing half of the number of sessions which is 2 for Adapted and 4 for Standard.

**Figure 2:**
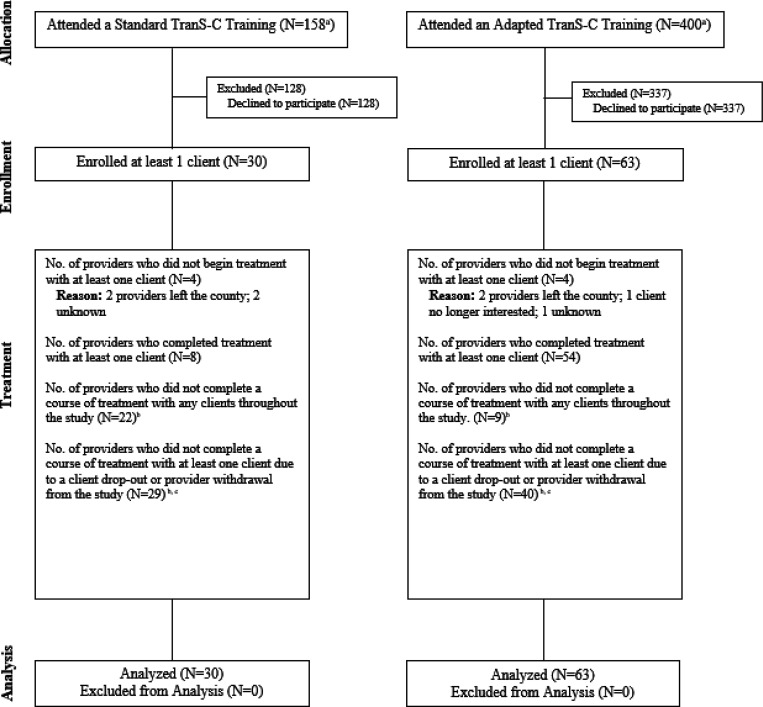
CONSORT Diagram Illustrating the Flow of Providers Through the Study *Note.* ^a^The larger N in Adapted vs. Standard TranS-C was a result of stronger recruitment in the counties randomized to this condition. ^b^As providers often treated multiple clients, the reason for non-completion varied based on the client or the timing of the provider’s departure from the county. Reasons for providers not completing a course of treatment included the provider leaving the county or study, clients no longer interested, and unknown factors. (See Figure 1 for more details about treatment dropouts). ^c^As providers were often matched with more than one client, the categories listed may overlap and thus do not sum to the total number of enrolled providers

See Fig. 1 for the CONSORT diagram for patients which includes, per the protocol paper, dropout rates at different stages of the trial. Dropout rates for participants in the immediate TranS-C condition were 14.14% (28 participants) before session 1, 34.48% (69 participants) between session 1 and the post-treatment assessment, and 4.04% (8 participants) between post and 6FU. Attrition rates were significantly higher in Standard than Adapted TranS-C during the treatment phase (50% in Standard; 25.81% in Adapted; χ^2^ = 11.00, df = 1, *p* < 0.001), but not significantly different prior to Session 1 (16.22% in Standard; 12.90% in Adapted; χ^2^ = 0.19, df = 1, *p* = 0.70), or at 6FU (4.05% in Standard; 4.03% in Adapted; χ^2^ < 0.01, df = 1, *p* = 1.00). Relative to completers, participants who did not begin treatment or who dropped out were not significantly different on stratification factors: sex (χ^2^ = 1.30, df = 1, *p* = .30), age group (above or below 50 years; χ^2^ = 0.72, df = 1, *p* = 0.40) or psychosis status (χ^2^ = 0.06, df = 1, *p* = 0.80). See Fig. 2 for the CONSORT diagram for providers.

As evident in Table 1, Standard and Adapted TranS-C did not differ on any pre-treatment patient demographic variable except on government assistance (*p* = 0.05) and education (*p* = 0.07), both of which approached significance. Specifically, more patients in Adapted had completed or had some graduate school education (8.06%) than in Standard (1.35%). Additionally, more patients in Standard reported using Medicaid (37.84%) than in Adapted (23.39%), while a greater number in Adapted were receiving Supplemental Social Security Income/Social Security Disability Insurance (SSI/SSDI) (25.81%) than in Standard (12.16%). As evident in Table 2, there were no significant differences in demographics between providers in Standard versus Adapted TranS-C. Additional File 4, Table 5 presents the patient demographics by treatment condition and immediate TranS-C vs. UC-DT which did not differ on any pre-treatment patient demographic variable except education (*p* = 0.07) approached significance.

Only four participants (2.02%) reported having potentially received a part of TranS-C during the UC-DT waiting period (e.g., “provider gave suggestions to alleviate anxiety before bed”, “completed one module”, “sleep diary with sleep therapist”). We deemed this to indicate minimal contamination.

### Aim 1

See Tables 3 and 4. TranS-C, relative to UC-DT, was significantly associated with improvements from pre- to post-treatment in sleep disturbance, sleep-related impairment, sleep health composite, psychiatric symptoms, and overall functional impairment. Sleep disturbance (primary outcome) withstood the Benjamini-Hochberg correction.

See Table 5 for Aim 1 SEM results. The indirect effect of sleep disturbance on the relations between treatment condition (TranS-C vs. UC-DT) and psychiatric symptoms approached significance. When county was included as covariate, the indirect effect of sleep disturbance on the relations between treatment condition (TranS-C vs. UC-DT) and psychiatric symptoms was significant (−2.49, 95% CI [−4.56, 0.43], MP = 36.44%). The indirect effect of sleep disturbance on the relations between treatment condition on overall functional impairment was significant. Similarly, the indirect effects of sleep-related impairment on the relations between treatment condition (TranS-C vs. UC-DT) and psychiatric symptoms and overall functional impairment were significant. The indirect effects explained 27.76–70.30% of the total effects in these models.

### Aim 2

See Table 6 for Aim 2 MLM results. TranS-C condition (Standard versus Adapted) was not significantly associated with providers’ perceptions of acceptability, feasibility, or appropriateness at post-treatment, accounting for pre-treatment.

### Aim 3

See Additional File 4, Table 6 for path coefficients and standard errors of Aim 3 SEMs. There were no significant indirect effects of acceptability on the relations between TranS-C condition (Adapted versus Standard) and the primary or secondary patient outcomes of sleep disturbance, sleep-related impairment, psychiatric symptoms, or overall functional impairment. The indirect effects explained 3.13% or less of the total effects for each of these models. This pattern of results held for appropriateness and feasibility, such that the indirect effects explained 15.23% or 11.16% or less of the total effects, respectively.

### Exploratory Aims

See Additional File 4, Table 7 for means and effect sizes of exploratory outcomes by timepoint and treatment condition and Additional File 4, Table 8 for MLM results.

For Exploratory Aim 1, Adapted TranS-C, relative to Standard TranS-C, was associated with a significant decrease in number of cigarettes per day from pre-treatment to six-month follow-up (*b* = −7.57, *p* = 0.005, *d* = −0.58). There were no significant differences in suicidal ideation severity, average daily caffeine use, or past 30-day alcohol use (all *p*s > .10).

There were no differences between Adapted versus Standard TranS-C on credibility (*b* = 0.14, SE = 0.32, *p* = 0.67, n_p_^2^ = 0.01), perceived improvement (*b* = 6.78, SE = 9.09, *p* = 0.46, n_p_^2^ = 0.02, or total CEQ (*b* = 0.10, SE = 0.18, *p* = 0.59, n_p_^2^ = 0.01). Looking across conditions, at post-treatment, the mean of the credibility items was 7.80 (*SD* = 1.52) and mean perceived improvement was 64.0% (*SD* = 28.57).

For Exploratory Aim 2, see Additional File 4, Table 9 for the MLM results. None of the planned demographics (sex, age) or baseline clinical symptoms (sleep disruption, sleep-related impairment, psychiatric symptoms) moderated the effects of treatment (UC-DT vs. TranS-C) on any of the primary or secondary patient outcomes (i.e., sleep disruption, sleep-related impairment, overall sleep health, psychiatric symptoms, overall functional impairment) from pre- to post-treatment (all *p*s > 0.10).

## Discussion

Consistent with the first hypothesis, relative to usual care and before delayed treatment, TranS-C (combining Adapted and Standard) was associated with reduced sleep disturbance, sleep-related impairment, sleep-health composite, psychiatric symptoms and functional impairment at the post-treatment assessment, relative to the pre-treatment assessment. This finding aligns with prior research that has used TranS-C for a range of different populations and non-CMHC settings (e.g., 74, 75, 76). Additionally, while these findings replicate a prior study conducted in a CMHC with SMI patients (18), they extend knowledge in two key areas. First, in the prior study, the providers were employed, trained and supervised within the university setting. In the present study, the providers were CMHC employees. Thus, the present study demonstrates that CMHC providers deliver TranS-C with positive outcomes despite carrying heavy and complex caseloads and working within the resource constraints of the CMHC context. As such, and following the NIH Stage Model (20), this study moves knowledge on TranS-C to Stage 3 by demonstrating “Efficacy in the Real World.” It is notable that a small group of patients in UC-DT (n = 4) were exposed to TranS-C during the waiting period, yet TranS-C was still more effective than UC-DT. Second, these results add to the existing evidence that a transdiagnostic treatment, designed to address a range of sleep and circadian problems experienced by a mixed diagnosis SMI sample, is helpful to patients seeking treatment in CMHCs (18). Indeed, Table 1 describes the broad range of diagnoses participants reported, with anxiety disorders, trauma-related disorders, mood disorders and schizophrenia spectrum disorders being most common.

Extending prior research showing that sleep treatment improves symptoms of comorbid mental health conditions (e.g., 17, 19, 77), TranS-C’s benefits for functional impairment and psychiatric symptoms were mediated by improvements in sleep and circadian problems. Re-stating this result in terms of the Experimental Therapeutics Approach (78, 79), we found evidence that TranS-C engaged the intended mechanism of change—*sleep and circadian processes—*which in turn, predicted change in patient outcomes.

The second hypothesis was that Adapted TranS-C, because it was adapted to improve fit, would be rated by CMHC providers as better fitted to the CMHC context relative to Standard TranS-C. There were no significant differences between the treatment conditions on the provider ratings of fit, including providers’ perception of acceptability, feasibility and appropriateness. In other words, the adaptation process we undertook—involving theory, data and end-user perspectives—resulted in a treatment that is shorter and simpler (four x 20-minute sessions) than the original version (eight x 50-minute sessions), while still attracting positive ratings from the providers. The finding that both Adapted and Standard TranS-C are acceptable to providers might be explained by the relative advantages of each approach. Specifically, Adapted TranS-C has the advantage that it is shorter and simpler and more easily delivered alongside the other interventions CMHC providers must deliver (e.g., housing support). Meanwhile, Standard TranS-C has the advantage that it more comprehensively treats a range of common sleep and circadian disorders. Interestingly, the lack of difference between the treatment conditions on provider ratings of fit suggests that the potential barriers to Standard TranS-C that we anticipated, such as time requirements and the complexity of the intervention, can be effectively managed in “real-life” routine practice settings. Given that many EBPTs follow a similar format to Standard TranS-C (i.e., multiple 50-minute sessions), this finding bodes well for the feasibility of scaling other EBPTs to settings akin to CMHCs. Overall, given the high provider ratings for both Adapted and Standard TranS-C, either form may be useful, with the specific choice depending on the characteristics of the setting and the patients.

Not surprisingly given the results just discussed and contrary to the third hypothesis, there were no significant effects for Adapted TranS-C to be associated with greater reductions in the primary and secondary patient outcomes indirectly via higher provider ratings of acceptability, feasibility and appropriateness, relative to Standard TranS-C. Although the hypothesized indirect effects were not significant, the mediation models did yield several interesting findings (Additional File 4, Table 6). For instance, the total effect of TranS-C condition on PROMIS-SD at post-treatment, as well as PROMIS-SRI at post-treatment and 6FU were significant in some of the mediation models, such that Adapted TranS-C, relative to Standard, was associated with improved sleep and circadian functioning. Also, higher provider ratings of fit was associated with better patient outcomes, regardless of the TranS-C condition. Although these are not prespecified analyses, such findings favor Adapted TranS-C and suggest that there may be differences worth exploring in future research.

For the exploratory aims, Adapted TranS-C was associated with a significant decrease in the number of cigarettes per day from pre-treatment to six-month follow-up (Pre: *M* = 11.18, *SD* = 8.3; 6FU: *M* = 6.78, *SD* = 6.6), relative to Standard TranS-C (Pre: *M* = 8.83, *SD* = 7.38; 6FU: *M* = 9.23, *SD* = 8.95). While the mean number of cigarettes per day for Adapted TranS-C was higher at the pre-treatment assessment, relative to the Standard TranS-C, the reduction by 4.4 cigarettes per day in Adapted is not trivial and adds another angle to existing evidence showing associations between smoking cigarettes and poor sleep (e.g., 80). Finally, there were no differences between Adapted versus Standard TranS-C on patient ratings of the credibility of the treatment or patient’s perceived improvement.

Overall, there are several signals that Adapted TranS-C may confer some advantages over Standard TranS-C. As already mentioned, findings within the mediation models and the number of cigarettes per day favored Adapted TranS-C. Also, the larger number of providers and patients in Adapted TranS-C (Providers = 63; Patients = 124) was a result of stronger recruitment in the counties randomized to this condition, as compared to Standard TranS-C (Providers = 30; Patients = 74). It is possible that Adapted TranS-C was more appealing to providers and patients. Moreover, there were significantly more drop-outs from Standard TranS-C relative to Adapted TranS-C during the treatment phase (Standard 50% vs. Adapted 25.81%), also possibly reflecting that Adapted TranS-C is more acceptable (81). Finally, in a separate qualitative study involving interviews with a subset of providers from the current study, providers expressed positive feedback for both versions of the treatment. However, for many outcomes, they showed a preference for Adapted TranS-C, while highlighting more unfavorable aspects of Standard TranS-C (82).

The present findings are consistent with the accumulating evidence indicating that facilitation is an effective approach that promotes the implementation of complex EBPTs, like TranS-C, into routine practice (e.g., 43, 44, 45). In the study described here—and consistent with the PARiHS framework (47) and guidelines (48, 49)—facilitation unfolded flexibly and differently depending on the unique challenges and obstacles faced by each site and each provider. There were many opportunities for support which engaged some but not all providers. Less popular offerings by the facilitation team included weekly drop-in supervision and as-needed consultation. More popular were presentations on advanced topics related to sleep and mental health (e.g., Lunch & Learn, Coffee Colloquium), setting up Continuing Education credits and working toward sleep treatment certification that CMHC providers could achieve via three supervised TranS-C cases. Preliminary evidence from the facilitation log that was collected throughout this study indicated that facilitators spent most of their time on internal team meetings for ongoing problem solving and sharing updates; simplifying and clarifying communication with CMHC providers about research-related logistics and study administration; preparing training materials, including making training dynamic; and delivering trainings and workshops (83).

Several notes and limitations warrant consideration. First, as specified in the protocol paper (25), the outcomes from this multi-site, multi-year study will be reported across multiple publications. This study has addressed the pre-specified outcomes for the main aims of the Implementation Phase. Future publications will focus on the Train-the-Trainer and Sustainment Phases. Other important outcomes—such as facilitation tracking—were not pre-specified and warrant separate in-depth publications. Second, there are several aspects that might seem like limitations but are, in fact, essential elements of this study. Specifically, although we trained providers to deliver 4 sessions in Adapted TranS-C and 8 sessions in Standard TranS-C, on the ground the Adapted providers delivered a mean of 4.99 sessions (SD = 1.91) and Standard providers delivered a mean of 8.95 sessions (SD = 8.01). This raises the possibility that providers may have believed patients needed more sessions than were originally prescribed. Also, due to the imperative to alleviate patient burden in this large multi-phase and multi-site study, patient self-reported psychiatric diagnoses were collected instead of administering a structured clinical interview. Similarly, it was not realistic to collect sleep diary and actigraphy. While sleep diary and actigraphy are gold standard measures for sleep research, they are not considered “essential” for “treatment studies focusing on large community samples in routine practice settings” (p. 1156; 84). Third, the design of the study precluded a comparison between conditions for the main outcomes at the 6-month follow-up. Also, we acknowledge that the UC-DT design may inflate the effect size differences (85) and that corrections for multiple secondary outcomes were not conducted. The secondary outcomes are not considered confirmatory, replication is needed. Fourth, while the present study monitored adverse events, adverse events should be pre-specified, pre-defined and measured (86). This is particularly vital in sleep treatments as adverse events have been documented (86). Fifth, the SDS is scored by summing the responses. However, participants who did not work or attend school skipped item 1: “The symptoms have disrupted your work/school.” Because higher SDS scores reflect greater impairment, the omission of this item may have artificially lowered the SDS scores. Also, the measure of fit we employed involved the providers rating items on a 1 (completely disagree) to 5 (completely agree) scale. As evident in Table 1, the mean ratings were above 4.5. Thus, a ceiling effect may have precluded group differences from emerging. Future research to refine the measurement of fit in a non-burdensome way is recommended. Notably, if fit was indicated by administrative metrics, like recruitment success or provider participation rates (which may imply feasibility and acceptability of the treatment and potential uptake), Adapted TranS-C shows better fit than Standard TranS-C. Sixth, it is possible that baseline differences in government assistance and education may have influenced the results, although these differences only trended toward significance. One final limitation is that it was not possible to distinguish drop-outs due to a patient discontinuing treatment from other causes, such as providers not having time to deliver an adequate number of treatment sessions. Clearly differentiating causes of drop-outs will be essential for future research.

## Conclusions

Both the Adapted and Standard versions of TranS-C were associated with improvement in sleep, psychiatric symptoms and functional impairment. These findings add to the growing support for the Sleep Health Framework (e.g., 13, 87, 88) and the use of facilitation as the implementation strategy (e.g., 43, 44, 45). This study was conducted in a community mental health setting and TranS-C was delivered by busy CMHC providers. As such, the findings constitute a meaningful step toward bridging the large gap between research and routine practice. Importantly, the use of theory, data and end-user perspectives to guide the adaptation of TranS-C responds to the surge of interest in increasing the scientific rigor of the treatment adaptation process (23, 30, 89) and yielded a range of interesting insights including that both Adapted and Standard TranS-C are valuable approaches, with some advantages favoring Adapted TranS-C.

Overall, the fact that Adapted TranS-C is equally effective and similarly rated as acceptable, feasible, and appropriate compared to Standard TranS-C is promising for supporting the use of a briefer version in under-resourced settings. The extent to which these findings are specific to CMHCs or generalizable to other contexts should be evaluated in future research.

## Supplementary Material

Supplement 1

## Figures and Tables

**Table 1.** Patient Demographics and Number of Sessions by Treatment Condition (Standard versus Adapted TranS-C) at Pre-Treatment

Characteristic	Standard TranS-C (*n* = 74)		Adapted TranS-C (*n* = 124)			
	*n*	*%*	*n*	*%*	c^2^	*p*-value
Sex					3.43	0.18
Female	42	56.76	83	66.94		
Male	31	41.89	41	33.06		
Missing/declined to answer	1	1.35	0	0.00		
Ethnicity					0.79	0.67
Hispanic or Latino	22	29.73	44	35.48		
Not Hispanic or Latino	51	68.92	79	63.71		
Missing/declined to answer	1	1.35	1	0.81		
Race					3.97	0.78
American Indian/Alaska Native	9	12.16	13	10.48		
Native Hawaiian/Pacific Islander	3	4.05	2	1.61		
Asian	7	9.46	7	5.65		
Black or African American	5	6.76	13	10.48		
White	40	54.05	68	54.84		
More than one race	4	5.41	6	4.84		
Other/category not listed	4	5.41	12	9.68		
Missing/declined to answer	2	2.70	3	2.42		
Education					8.50	0.07
High school graduate or below	21	28.38	40	32.26		
Some or completed college or vocational school	50	67.57	73	58.87		
Some or completed graduate school	1	1.35	10	8.06		
Other/category not listed	0	0.00	1	0.81		
Missing/declined to answer	2	2.70	0	0.00		
Employment					4.12	0.39
Full-time	11	14.86	16	12.90		
Part-time	12	16.22	26	20.97		
Not employed	46	62.16	78	62.90		
Other/category not listed	3	4.05	4	3.23		
Missing/declined to answer	2	2.70	0	0.00		
Civil Status					3.45	0.18
Partnered	12	16.22	19	15.32		
Unpartnered	60	81.08	105	84.68		
Missing/declined to answer	2	2.70	0	0.00		
Living Arrangement					7.41	0.19
Alone	9	12.16	31	25.00		
With family	49	66.22	66	53.23		
With friend or roommate or pet	11	14.86	17	13.71		
Supportive housing	2	2.70	6	4.84		
Other/category not listed	2	2.70	4	3.23		
Missing/declined to answer	1	1.35	0	0.00		
Government Assistance^a^					17.10	0.05
Unemployment	6	8.11	4	3.23		
Medicare	7	9.46	22	17.74		
Medicaid	28	37.84	29	23.39		
Social Security	8	10.81	14	11.29		
Food Stamps	25	33.78	34	27.42		
SSI/SSDI	9	12.16	32	25.81		
SNAP	2	2.70	4	3.23		
None	0	0.00	4	3.23		
Other/category not listed	7	9.46	21	16.94		
Missing/declined to answer	20	27.03	29	23.39		
Annual Personal Income					6.84	0.45
<$10,000	18	24.32	39	31.45		
$10,000-$20,000	21	28.38	36	29.03		
$20,000-$30,000	8	10.81	12	9.68		
$30,00-$40,000	4	5.41	4	3.23		
$40,000-$50,000	1	1.35	5	4.03		
>= $50,000	2	2.70	7	5.65		
I don’t know my income	19	25.68	21	16.94		
Missing/declined to answer	1	1.35	0	0.00		
Annual Household income					6.80	0.45
<$10,000	10	13.51	23	18.55		
$10,000-$20,000	16	21.62	31	25.00		
$20,000-$30,000	11	14.86	10	8.06		
$30,00-$40,000	4	5.41	6	4.84		
$40,000-$50,000	2	2.70	6	4.84		
>= $50,000	6	8.11	18	14.52		
I don’t know my income	24	32.43	28	22.58		
Missing/declined to answer	1	1.35	2	1.61		
Self-reported diagnosis^b^					9.62	0.65
Neurodevelopmental disorders	8	10.81	9	7.26		
Psychosis	23	31.08	40	32.26		
Bipolar Disorder	22	29.73	29	23.39		
Major Depressive Disorder	43	58.11	54	43.55		
Anxiety disorders	38	51.35	73	58.87		
Obsessive-compulsive and related disorders	2	2.70	6	4.84		
Trauma and stressor-related disorders	24	32.43	35	28.23		
Dissociative disorders	1	1.35	3	2.42		
Personality disorders	3	4.05	3	2.42		
Feeding and eating disorders	2	2.70	2	1.61		
Substance-related and addictive disorders	2	2.70	1	0.81		
Other/category not listed	1	1.35	3	2.42		
Missing/declined to answer	1	1.35	9	7.26		
	*Mean*	*SD*	*Mean*	*SD*	t	*p*-value
Age	38.84	13.56	42.42	16.36	-1.65	0.10
Education (years)	13.59	2.87	13.87	3.35	-0.61	0.54
No. of sessions received (all)^c^	5.43	6.76	3.85	2.71	1.93	0.06
No. of sessions received (completers)^d^	8.95	8.01	4.99	1.91	2.97	< 0.01

*Note.* Chi-squared was used for categorical variables, and *t* tests were used for continuous variables.

a Some patients endorsed more than one government assistance category.

b Comorbidity was common.

c Number of TranS-C sessions received by all enrolled patients in the study.

d Number of TranS-C sessions received by patients who completed treatment.

**Table 2.** Provider Demographics by TranS-C Treatment Condition (Standard versus Adapted TranS-C) at Pre-Treatment

Characteristic	Standard TranS-C (*n* = 30)		Adapted TranS-C (*n* = 63)			
	*n*	*%*	*n*	*%*	c^2^	*p*-value
Sex					3.26	0.35
Female	23	76.67	47	74.60		
Male	5	16.67	5	7.94		
Other/category not listed	0	0.00	1	1.59		
Missing/declined to answer	2	6.67	10	15.87		
Ethnicity					0.09	0.95
Hispanic or Latino	9	30.00	17	26.98		
Not Hispanic or Latino	15	50.00	33	52.38		
Missing/declined to answer	6	20.00	13	20.63		
Race					5.11	0.53
American Indian/Alaska Native	0	0.00	2	3.17		
Asian	3	10.00	7	11.11		
Native Hawaiian or Pacific Islander	0	0.00	2	3.17		
Black or African American	2	6.67	1	1.59		
White	19	63.33	32	50.79		
More than one race	1	3.33	5	7.94		
Missing/declined to answer	5	16.67	14	22.22		
Degree Type					10.08	0.18
Marriage and Family Therapy	10	33.33	13	20.63		
Psychology	3	10.00	7	11.11		
Social Work	8	26.67	20	31.75		
Nursing	0	0.00	1	1.59		
Medical	1	3.33	0	0.00		
Occupational Therapy	0	0.00	5	7.94		
Other/category not listed	7	23.33	5	7.94		
Missing	4	13.33	12	19.05		
Therapeutic Approach^a^					9.45	0.22
Client Centered	19	63.33	40	63.49		
Family Systems	5	16.67	15	23.81		
CBT	21	70.00	30	47.62		
Psychodynamic	7	23.33	11	17.46		
Humanistic	2	6.67	0	0.00		
Integrative/Holistic	1	3.33	3	4.76		
None	0	0.00	3	4.76		
Missing/declined to answer	3	10.00	13	20.63		
Licensure					0.92	0.63
Licensed	16	53.33	32	50.79		
Not Licensed	11	36.67	20	31.75		
Missing/declined to answer	3	10.00	11	17.46		
	*Mean*	*SD*	*Mean*	*SD*	t	*p*-value
Age	38.26	10.06	41.54	11.38	0.97	0.34
Caseload	40.09	23.97	29.59	31.62	1.47	0.15
Employment Duration	3.87	3.51	3.84	3.96	0.03	0.98
Years Since Degree Earned	9.44	7.66	9.62	7.40	−0.09	0.93

Note.

a Some providers endorsed more than one therapeutic approach. Chi-squared was used for categorical variables, and *t* tests were used for continuous variables. CBT = cognitive behavioral therapy. Caseload = number of clients on caseload. Employment duration = length of time employed at current CMHC in years.

**Table 3.** Means, Standard Deviations, and Effect Sizes for Primary and Secondary Outcomes

	Pre-Treatment				Post-Treatment				
Patient Outcomes									
	UC-DT		TranS-C		UC-DT		TranS-C		*d*
	Mean	SD	Mean	SD	Mean	SD	Mean	SD	
PROMIS-SD*	62.80	7.89	62.76	7.15	61.65	8.33	50.83	10.07	−1.52
PROMIS-SRI	62.01	8.07	62.06	8.82	61.17	8.42	51.78	10.38	−1.06
SHC	2.07	1.33	1.99	1.46	2.22	1.49	3.54	1.55	0.95
DSM-5	24.18	9.43	24.3	8.97	23.35	8.82	18.88	10.62	−0.52
SDS	13.15	6.72	12.63	7.38	12.36	7.19	6.49	6.06	−0.71
Provider Outcomes									
	Standard TranS-C		Adapted TranS-C		Standard TranS-C		Adapted TranS-C		*d*
	Mean	SD	Mean	SD	Mean	SD	Mean	SD	
AIM*	4.69	0.41	4.70	0.46	4.64	0.63	4.67	0.53	0.06
FIM	4.62	0.46	4.61	0.48	4.61	0.74	4.61	0.52	0.02
IAM	4.62	0.5	4.69	0.46	4.59	0.69	4.59	0.54	−0.16

* indicates primary outcome. Effect sizes are represented with ‘*d*’ and were calculated following Feingold (2009, equation 5), using unadjusted change scores and raw standard deviations at pre-treatment from each treatment condition. PROMIS-SD = PROMIS Sleep Disruption. PROMIS-SD = PROMIS Sleep Disturbance. PROMIS-SRI = PROMIS Sleep-Related Impairment. SHC = Sleep Health Composite (note, scored such that higher scores indicate better sleep health). DSM-5 = DSM-5 Cross-Cutting. SDS = Sheehan Disability Scale. AIM = Acceptability of Intervention Measure. FIM = Feasibility of Intervention Measure. IAM = Intervention Appropriateness measure. TranS-C = Transdiagnostic Intervention for Sleep and Circadian Dysfunction. UC-DT = usual care followed by delayed treatment with TranS-C.

**Table 4.** Aim 1: Multilevel Modeling Results for Treatment Condition (UC-DT versus TranS-C) on Patient Outcomes from Pre- to Post-Treatment

	*b*	SE	*p*-value
**Outcome**			
PROMIS-SD	−10.91	1.94	**< 0.001**
PROMIS-SRI	−9.52	1.95	**< 0.001**
SHC	1.63	0.35	**< 0.001**
DSM-5	−6.72	1.46	**< 0.001**
SDS	−5.12	1.34	**< 0.001**

*Note.* Bold indicates significant *p*-values. *b* = time-by-treatment interaction. SE = robust standard errors. PROMIS-SD = PROMIS Sleep Disturbance. PROMIS-SRI = PROMIS Sleep-Related Impairment. SHC = Sleep Health Composite. DSM-5 = DSM-5 Cross-Cutting. SDS = Sheehan Disability Scale.

**Table 5.** Aim 1: Mediation Models of Sleep Outcomes on Relations between Treatment Condition (TranS-C vs. UC-DT) and Psychiatric Symptoms and Overall Functional Impairment at Post-Treatment

	coefficient	SE	z	*p*	95% Confidence Interval of effect	%MP
**Aim 1 Model 1: TranS-C vs. UC-DT à PROMIS-SD à DSM-5 POST**						
Path a	−12.40	1.66	−7.48	<0.001	−15.65, −9.15	-
Path b	0.15	0.08	1.88	0.06	−0.01, 0.31	-
Total effect	−6.70	1.26	−5.31	<0.001	−9.17, −4.23	-
Indirect effect	−1.86	1.05	−1.76	0.08	−3.93 −0.21	27.76%
**Aim 1 Model 2: TranS-C vs. UC-DT à PROMIS-SD à SDS Post**						
Path a	−12.38	1.66	−7.44	<0.001	−15.64, −9.12	-
Path b	0.25	0.06	4.45	<0.001	0.14, 0.36	-
Total effect	−5.27	1.17	−4.51	<0.001	−7.56, −2.98	-
Indirect effect	−3.12	0.74	−4.20	**<0.001**	**−4.58, −1.66**	59.20%
**Aim 1 Model 3: TranS-C vs. UC-DT à PROMIS-SRI à DSM-5 POST**						
Path a	−10.59	1.79	−5.92	<0.001	−14.09, −7.08	-
Path b	0.24	0.08	2.95	0.003	0.08, 0.39	-
Total effect	−6.94	1.26	−5.49	<0.001	−9.41, −4.46	-
Indirect effect	−2.51	1.06	−2.37	**0.02**	**−4.58, −0.44**	36.17%
**Aim 1 Model 4: TranS-C vs. UC-DT à PROMIS-SRI à SDS POST**						
Path a	−10.60	1.81	−5.85	0.001	−14.14, −7.05	-
Path b	0.36	0.05	7.42	<0.001	0.26, 0.46	-
Total effect	−5.42	1.17	−4.62	<0.001	−7.72, −3.12	-
Indirect effect	−3.81	0.81	−4.69	**< 0.001**	**−5.41, −2.22**	70.30%

*Note.* Significant effects for parameters of primary interest (i.e., indirect effects) are bolded. “-”indicates that value is not relevant to model. SE = robust standard errors. %MP = mediated proportion(i.e., the proportion of the total effect that is explained by the indirect effect expressed as a percentage). TranS-C = Transdiagnostic Intervention for Sleep and Circadian Dysfunction. UC-DT =usual care followed by delayed treatment with TranS-C. PROMIS-SD = PROMIS Sleep Disturbance. PROMIS-SRI = PROMIS Sleep-Related Impairment. SDS = Sheehan Disability Scale. DSM-5 = DSM-5Cross-Cutting. POST = post-treatment assessment. Path a = path from the independent variable to mediator (i.e., Treatment condition à PROMIS-SD or PROMIS-SRI). Path b = path from the mediator to the outcome (PROMIS-SD or PROMIS-SRI à DSM-5 Cross-Cutting or SDS). All models adjusted for pre-treatment levels of the relevant mediator (i.e., PROMIS-SD or PROMIS-SRI) and relevant outcome (i.e.,DSM-5 Cross-Cutting or SDS).

**Table 6.** Aim 2: Multilevel Modeling Results for TranS-C Condition (Adapted vs. Standard) on Provider Perceptions of Treatment Fit from Pre- to Post-Treatment

	*b*	SE	*p* value
**Outcome**			
AIM	−0.03	0.11	0.77
FIM	−0.01	0.14	0.94
IAM	−0.11	0.12	0.34

*Note. b* = time-by-treatment interaction. SE = robust standard errors. AIM = Acceptability of Intervention Measure. FIM = Feasibility of Intervention Measure. IAM = Intervention Appropriateness measure.

## Data Availability

Raw data for most outcomes reported herein has been uploaded into the NIMH National Data Archive.

## Footnotes

1. Note that the vast majority of providers and patients were employed by or seeking/receiving treatment at CMHCs. However, in very few isolated instances, providers and patients outside of CMHCs learned about the study (e.g., by word of mouth) and requested to participate. When the providers or patients otherwise met the criteria, they were permitted to participate, and were matched with a CMHC patient or provider, respectively, by the facilitation team.

2. This approach was taken when the patient and provider were uncertain about the SMI diagnosis.

3. Due to administrative error, the 16-item version was originally administered. However, standardized T-scores are only available for the 8-item version. Thus, only the 8 items that comprise the 8-item version were included in the scoring and analyses.

4. This approach to mediation deviates from the approach described in the protocol paper (i.e., multilevel SEMs). This change was made, because there is limited published guidance on using multilevel SEM for mediation in longitudinal RCTs. Although our syntax for multilevel SEMs produced the same pattern of results as those produced with the ANCOVA approach, we felt more confident in the estimates yielded for the latter.

## Abbreviations

6FU: six months after treatment
AIM: Acceptability of Intervention Measure
ANCOVA: analysis of covariance
CBT: cognitive behavioral therapy
CEQ: Credibility/Expectancy Questionnaire
CMHCs: community mental health centers
DSM: 5-DSM-5 Cross-Cutting Measure
EBPT: evidence-based psychological treatments
FIM: Feasibility of Intervention Measure
IAM: Intervention Appropriateness measure
ICC: intraclass correlation coefficients
MDES: minimum detectable effect sizes
MLMs: multilevel models
MP: mediated proportions
PROMIS: SD-PROMIS-Sleep Disturbance
PROMIS: SRI-PROMIS-Sleep Related Impairment
REP: Replicating Effective Programs
SDS: Sheehan Disability Scale
SE: robust standard errors
SEMs: structural equation models
SHC: Sleep Health Composite
SMI: serious mental illness’
SNAP: Supplemental Nutrition Assistance Program
SSI/SSDI: Supplemental Social Security Income/Social Security Disability Insurance
UC: DT-Usual Care followed by Delayed Treatment with TranS-C
