# Supplementary material for: A randomized controlled trial of the Transdiagnostic Intervention for Sleep and Circadian dysfunction implemented via facilitation and delivered by community mental health providers: Improving the “fit” of psychological treatments by adapting to context
Source: Res Sq. 2024 Dec 24:rs.3.rs-5422372. Preprint. [Version 1] doi: 10.21203/rs.3.rs-5422372/v1 (PMC11703346; doi:10.21203/rs.3.rs-5422372/v1)
Supplement: Supplement 1 [file NIHPPRS5422372V1-supplement-1.pdf]

Supplementary Files

This is a list of supplementary files associated with this preprint. Click to download.

- Gen1AdditionalFilesFinalFINALtham3.docx
- StaRIchecklistHarvey.docx
